# F. H. Rehwinkel

**Published:** 1889-07

**Authors:** 


### Obituary.

---

F. H. REHWINKEL, M. D., D. D. S.—Died, at Chillicothe, Ohio, June 8th, 1889, of Paralysis, F. H. Rehwinkel, M. D., D. D. S., in the sixty-fourth year of his age.

Dr. Rehwinkel was born at Celle, Hanover, Germany, June 15th, 1825, and in that city began his medical education, which was afterwards completed at a German university. He came to this country in 1849, commenced medical practice at Natchez, Tenn., and subsequently was associated with Dr. J. H. Pulte, in Cincinnati, Ohio. In 1850 he removed to Chillicothe, and was married on the 25th of November in that year. He opened an office in Portsmouth, Ohio, but a year afterwards returned to Chillicothe, and about the year 1853 turned his attention to dentistry, and graduated from the Baltimore College of Dental Surgery in the year 1854, since which time he has practiced dentistry uninterruptedly with the exception of the years 1861 and 1862, during which he was with the Union army. After a period of severe service he went home on sick leave, and on his way to rejoin his regiment met with an accident which necessitated nearly a year's use of crutches and compelled him to leave the service. About one year ago, while hurrying to catch a train he was overcome with the heat, and has since been in precarious health, although his physicians did not anticipate the sudden and fatal shock. His wife and a daughter survive him.

The funeral obsequies of Dr. Rehwinkel brought to Chillicothe a large company of distinguished men, including the governor of the State. The ceremonies are said to have been the most impressive and imposing ever witnessed in that city, which fact sufficiently attests the high regard in which he was held. His professional associates and friends will no less publicly and warmly manifest their respect and affection in letters and resolations of commendation and condolence.

Dr. Rehwinkel's connection with and activity in State and National dental and medical associations are very well known. He has been president of the Ohio State Dental Society, Mississippi Valley Dental Association, and the American Dental Association; secretary of the Dental Section of the Ninth International Medical Congress; chairman of the section of Oral and Dental Surgery of the American Medical Association, etc. His contributions to dental literature have been considerable, and an intimate acquaintance with European dentists of eminence enabled him to introduce them and their works to the profession in America. He was always modestly prominent in the meetings of every dental society which he could by any means attend, and his demeanor under all circumstances was such as to win and hold the confidence and affection of his professional brethren. His skill in practice was of a high order, and his character was that of the honored and honorable citizen, patriot, practitioner, friend, husband, and father who can be eulogized without hesitation or mental reservation. No man in the dental profession of America was more universally trusted and beloved than he, and the death of no one could be more sincerely lamented.

---

To the above, which is taken from the *Cosmos*, we desire to add that Dr. Rehwinkel was a fellow-student and classmate of the editor of this Journal, who is the only survivor of the six students who boarded together in Baltimore during our course at the Baltimore College. All that the above notice commends in our deceased classmate and friend, we heartily endorse, and rejoice that he afforded an example which all may follow with benefit to themselves and honor to their profession.

